# Accelerating Smith-Waterman Alignment for Protein Database Search Using Frequency Distance Filtration Scheme Based on CPU-GPU Collaborative System

**DOI:** 10.1155/2015/761063

**Published:** 2015-10-19

**Authors:** Yu Liu, Yang Hong, Chun-Yuan Lin, Che-Lun Hung

**Affiliations:** ^1^School of Electronic Information Engineering, Tianjin University, Tianjin 300072, China; ^2^Department of Computer Science and Information Engineering, Chang Gung University, Taoyuan 33302, Taiwan; ^3^Department of Computer Science and Communication Engineering, Providence University, Taichung 43301, Taiwan

## Abstract

The Smith-Waterman (SW) algorithm has been widely utilized for searching biological sequence databases in bioinformatics. Recently, several works have adopted the graphic card with Graphic Processing Units (GPUs) and their associated CUDA model to enhance the performance of SW computations. However, these works mainly focused on the protein database search by using the intertask parallelization technique, and only using the GPU capability to do the SW computations one by one. Hence, in this paper, we will propose an efficient SW alignment method, called CUDA-SWfr, for the protein database search by using the intratask parallelization technique based on a CPU-GPU collaborative system. Before doing the SW computations on GPU, a procedure is applied on CPU by using the frequency distance filtration scheme (FDFS) to eliminate the unnecessary alignments. The experimental results indicate that CUDA-SWfr runs 9.6 times and 96 times faster than the CPU-based SW method without and with FDFS, respectively.

## 1. Introduction

In bioinformatics, the sequence alignment has become one of the most important issues. When the biologists get an unknown sequence, in general they would compare this unknown sequence (denoted as query sequence) with the known database of sequences (denoted as database sequences) to find the similarity scores and then identify the evolutionary relationships among them. Needleman and Wunsch [1] proposed a dynamic programming method (abbreviated to NW algorithm) to solve the global alignment problem between two sequences in 1970. The Smith-Waterman (abbreviated to SW) algorithm, which was proposed by Smith and Waterman [2] in 1981, is designed to find the optimal local alignment, and it is enhanced by Gotoh [3] in 1982. Although Hirschberg's algorithm [4] can be used for these algorithms above to reduce the memory space requirement, the computing time increases by a factor of two. When the lengths of two sequences are *m* and *n*, respectively, the time complexities of both NW and SW algorithms are *O*(*mn*), respectively, and their space complexities by adapting Hirschberg's algorithm can be both reduced from *O*(*mn*) to *O*(*m*), where *m* is assumed to be larger than *n*. Though the NW and SW algorithms guarantee the maximal sensitivity for the alignment, the cost is still expensive, especially for the computation time.

Several fast heuristic methods such as FASTA [5] and BLAST [6, 7] have been devised to reduce the computation time at the expense of sensitivity. However, the exponential increase in the number of known sequences has increased the search time for querying against a database. Recently many core architectures, such as FPGA [8–10], Cell/Bes [11–13], and Graphics Processing Units (abbreviated to GPUs) [14, 15], have gradually become more popular in bioinformatics. These new architectures make it possible to enhance the performance of sequence alignments by using the parallel computing technologies. The use of GPUs has gradually mainstreamed in the high-speed computing field. The potential advantage of GPU is its thousands of cores, with their total computing power exceeding the architecture with few CPUs. Also NVIDIA released the compute unified device architecture (abbreviated to CUDA) model to allow the programmers to use the commonly used programming languages, such as C/C++, to develop the applications. Thus many efforts have been made to accelerate the SW computations by CUDA on GPU.

In 2008, Manavski and Valle [16] presented the first SW algorithm by CUDA for the protein database search on GPU. The proposed algorithm was enhanced by the GSW algorithm proposed by Striemer and Akoglu [17] in 2009. Ligowski and Rudnicki [18] also presented another SW algorithm for the protein database search on GPU in 2009. Liu et al. proposed CUDASW++1.0 [19] and CUDASW++2.0 [20] for protein database search in 2009 and 2010, respectively. In CUDASW++1.0, they defined the intertask parallelization (abbreviated to ITE) and intratask parallelization (abbreviated to ITR) techniques for the relationships of task-thread and task-thread block on GPU, respectively. In general, the performance of the ITE technique is better than that of the ITR technique; however the ITE technique requires more memory space and then it is suitable for short sequences. Khajeh-Saeed et al. [21] proposed a CUDA-based SW algorithm (abbreviated to CUDA-SSCA#1) by using the ITR technique and multiple GPUs in 2010. In 2011, Hasan et al. [22] proposed a GPU-based SW algorithm (abbreviated to HKA algorithm) for the protein database search by using new sequence database organization and several optimizations to reduce the number of memory accesses. Hains et al. [23] developed new ITE and ITR kernels of SW algorithm for the protein database search in 2011. Sandes and de Melo proposed CUDAling1.0 [24] and CUDAling2.0 [25] for comparing two huge genomic sequences on GPU in 2010 and 2011, respectively. Liu et al. [26] presented CUDASW++3.0 in 2013 for the protein database search by coupling the CPU and GPU SIMD instructions and doing the CPU and GPU computations concurrently.

With the development of next-generation sequencing (NGS) techniques, the NGS machines can generate more than 1000 million nucleotide short reads (DNA, mRNA, and small-RNA) of lengths around 30~50 bps or more in a single run. For several NGS applications, such as metagenomics, these short reads will be assembled into possible contigs with lengths of tens to hundreds. These contigs are then used to match several well-known databases, such as NCBI-nt database, in order to classify or filter out these contigs. For coding DNA or mRNA short reads, they also can be translated to proteins for the proteomic research or be used for the analysis procedure of transcriptome. Now, several databases have more than hundreds of thousands protein (or nucleotide) sequences with lengths of tens to hundreds (or hundreds to tens of thousands). However, the previous works mentioned above are not suitable for the protein database search with a lot of database sequences. There are two reasons. One is that most of these works are suitable for short query and database sequences by using the ITE technique. The other is that these works all do the SW computations one by one (seen as the brute force search), and the computation time will be large for comparing a query sequence with a lot of database sequences under the limited hardware resources. Therefore, it is important to provide new concepts and procedures for the protein database search problem.

There is a possible method to compare a query sequence with a lot of database sequences, called CUDA-SWf proposed by Lee et al. [27] in 2013. CUDA-SWf used the frequency distance filtration scheme (abbreviated to FDFS) [28] in the run-time to filter out the unnecessary alignments, and then the computation time by CUDA-SWf is improved up to 41%. However, CUDA-SWf is also designed by using the ITE technique. The FDFS in CUDA-SWf is to calculate the frequency vectors of query and database sequences on GPU at first; then it calculates the frequency distance for each pair of query and database sequences, and after that the database sequences that need to be compared (denoted as selected database sequences) should be transferred from GPU to CPU. Finally, these selected database sequences are sorted according to their lengths and then retransferred from CPU to GPU in order to do the SW computations. Hence, the computation time by CUDA-SWf may be large for a lot of database sequences.

In this paper, we will propose an efficient SW alignment method, called CUDA-SWfr, for the protein database search by using the ITR technique based on a CPU-GPU collaborative system. In order to avoid the unnecessary alignments, FDFS is also applied to CUDA-SWfr to enhance the computation performance. For most of bioapplications, the used database can be predownloaded and preprocessed according to the application requirements. Therefore, the frequency vectors of database sequences can be precalculated and then stored in the database. Before doing the SW computations on GPU, FDFS is executed on CPU by calculating the frequency distances for each pair of frequency vectors from a query and database sequences. After that, the query and selected database sequences are transferred from CPU to GPU. The computation time of this procedure can be overlapped with that of SW computations. The experimental results indicate that CUDA-SWfr runs 9.6 times and 96 times faster than the CPU-based SW method without and with FDFS, respectively. These results indicated that CUDA-SWfr is suitable for the protein database search with a lot of database sequences.

The rest of this paper is organized as follows.  Section 2 briefly describes the background knowledge of CUDA-SWfr, including the SW algorithm, the CUDA programming model, the frequency vector and frequency distance, and the related works of SW algorithm by CUDA on GPU.  Section 3 then introduces CUDA-SWfr consisting of the implementations of FDFS on CPU and the SW computations on GPU.  Section 4 gives the experimental results to evaluate CUDA-SWfr without and with FDFS.

## 2. Background Knowledge

### 2.1. The SW Algorithm

The SW algorithm is designed to identify the optimal local alignment between two sequences (query and database sequences). The SW computation needs a substitution matrix, such as a series of BLOSUM [28] or PAM [29] matrices, and a gap-penalty function, such as the constant gap penalty or the affine gap penalty. The SW algorithm adopted in CUDA-SWfr is the same as that used in CUDA-SSCA#1 [21] with the affine gap penalty. Given two sequences *S*_1_ and *S*_2_ with lengths *l*_1_ and *l*_2_, respectively, the SW algorithm computes the similarity score in an alignment matrix *H*(*i*, *j*) of these two sequences ending at positions *i* and *j* of sequences *S*_1_ and *S*_2_, respectively. The alignment matrix *H*(*i*, *j*) is computed according to

(1) $$\begin{array}{c} H_{i,j}=\mathrm{Max}\left\{\begin{array}{cc} \mathrm{Max}\left(H_{i-1,j-1}+S_{ij},0\right) & \\ \underset{0<k<i}{\mathrm{Max}}\left(H_{i-k,j}-\left(G_{s}+kG_{e}\right)\right) & \\ \underset{0<k<j}{\mathrm{Max}}\left(H_{i,j-k}-\left(G_{s}+kG_{e}\right)\right), & \end{array}\right. \end{array}$$

where 1 ≤ *i* ≤ *l*_1_, 1 ≤ *j* ≤ *l*_2_, and *S*_*ij*_ is the score in a substitution matrix, which is extracted according to a residue at position *i* in sequence *S*_1_ and another residue at position *j* in sequence *S*_2_. *G*_*s*_ is the gap opening penalty, *G*_*e*_ is the gap extension penalty, and *k* is the number of the extended gaps.

**Figure 1:**
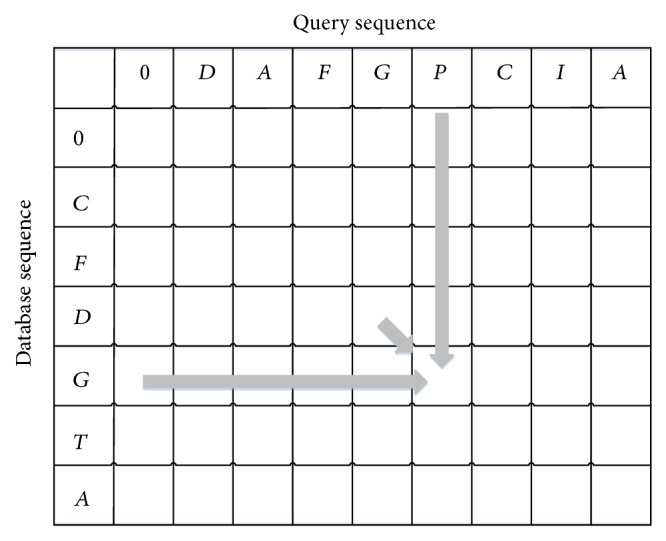
Dependency of calculating an alignment matrix *H*(*i*, *j*).

The maximum value of alignment matrix *H*(*i*, *j*) indicates the similarity score between two sequences. The dependency of calculating an alignment matrix *H*(*i*, *j*) is shown in Figure 1. As mentioned in the literature [21], formula (1) is the native concept of the SW algorithm. In order to improve the SW computation, the SW algorithm is modified as formula (2) according to the literature [3]. Formula (2) is more suitable for the parallel computing and the details of SW algorithm can be found in the literature [21]:

(2) Ei,j=Max⁡Ei,j−1,Hi,j−1−Gs−Ge,Fi,j=Max⁡Fi−1,j,Hi−1,j−Gs−Ge,Hi,j=Max⁡Hi−1,j−1+Sij,Ei,j,Fi,j,0.

### 2.2. CUDA Programming Model

CUDA is an extension of commonly used programming languages, such as C/C++, in which users can write scalable multithreading programs for various applications. In general, the CUDA program is implemented in two parts:* Host* and* Device*. The* Host* part is executed by CPU, and the* Device* part is executed by GPU. The function executed on the* Device* part is called a* Kernel*. The* Kernel* can be invoked as a set of concurrently executing threads (abbreviated to TDs). These TDs are grouped into a hierarchical organization which can be combined into thread blocks (abbreviated to TBs) and grids (abbreviated to GDs). A GD is a set of independent TBs, and a TB contains many TDs. The size of GD is the number of TBs per GD, and the size of TB is the number of TDs per TB.

The TDs in a TB can communicate and synchronize with each other. TDs within a TB can communicate through a per-TB shared memory (abbreviated to sM), whereas TDs in the different TBs fail to communicate or synchronize directly. Besides sM, five memory types are per-TD private local memory (abbreviated to LM), global memory (abbreviated to GM) for data shared by all TBs, texture memory (abbreviated to TM), constant memory (abbreviated to CM), and registers (abbreviated to RG). Of these memory types, CM and TM can be regarded as fast read-only caches; the fastest memories are the register and sM. The GM, LM, TM, and CM are located on the GPU's memory. Besides sM accessed by a single TB and RG only accessed by a single TD, the other memory types can be used by all TDs. The caches of TM and CM are limited to 8 KB per streaming multiprocessor (abbreviated to SM). In the Kepler architecture, SM is also called SMX. The optimum access strategy for CM is all TDs reading the same memory address. The cache of TM is designed for TDs in order to improve the efficiency of memory access. The Fermi and Kepler architectures have real configurable L1 per SM and unified L2 caches among SMs. Hence, L2 caches can be accessed by GM and each SM can use the L1 caches and sM.

The basic processing unit in NVIDIA's GPU architecture is called the streaming processor (abbreviated to SP). In the Fermi and Kepler architectures, the basic processing unit is called CUDA cores. Many SPs perform the computations on GPU. Several SPs can be integrated into a SM according to various architectures, such as 32 and 192 SPs per SM for the Fermi and Kepler architectures, respectively. While the program runs the* Kernel*, the* Device* schedules TBs for the execution on the SM. The Single Instruction Multiple Thread (abbreviated to SIMT) scheme refers to TDs running on the SM in a small group of 32, called a warp (abbreviated to WP). The WP scheduler simultaneously schedules and dispatches instructions.

### 2.3. Frequency Vector and Frequency Distance

The frequency vector and frequency distance are proposed by Kahveci et al. [30] in 2004, and they are used to the whole genome alignment problem. In the literature [31], the frequency distance is also used to remove the sequences which are dissimilar between the query and subject sequences before performing the sequence alignment. In 2013, Lee et al. [27] used the frequency vector and frequency distance to remove the unnecessary SW alignments between the query and database sequences. Assuming that a query or database sequence *s* is composed of *n* kinds of nucleotides/amino acids (denoted as the alphabet set), the frequency vector (abbreviated to FV) of *s* is defined as follows:

(3) $$\begin{array}{c} \text{FV}\left(\;s\;\right)=f_{s}=\left[\;\alpha_{1},\alpha_{2},\alpha_{3},\ldots ,\alpha_{n}\;\right], \end{array}$$

where *α*_*n*_ represents the number of *n*th alphabets appearing in the sequence *s*. Assume that there are two DNA sequences *u* and *v*; the frequency distance (abbreviated to FD) of these two sequences is defined as follows:

(4) FDfu,fv=fu−fv=Au−Av+Tu−Tv+Gu−Gv+Cu−Cv.

The FD is calculated from FVs of sequences *u* and *v*, which is the sum of differences for FVs of the alphabet set. For the biological sequences, the edit distance (abbreviated to ED) is a commonly used measurement to represent the difference between two sequences. In other words, a low edit distance means a high similarity score. The relation between FD and ED for sequences *u* and *v* is listed as follows:

(5) $$\begin{array}{c} \text{FD}\left(\;f_{u},f_{v}\;\right)\leq \text{ED}\left(\;u,v\;\right). \end{array}$$

In several bioapplications, a threshold as a lower bound of similarity score may be defined by the biologists to filter out the unwanted results. For example, in the homology modeling application, the similarity score should be larger than 35% between the template and target sequences. Moreover, in general, the sequence coverage ratio should be larger than 60% between the template and target sequences. Therefore, this threshold could be used as a factor to filter out unnecessary alignments between a query and database sequences. When we want to find the very similar sequences in a database for a query sequence, the value of ED is set to small. If a FD of a pair of query and database sequences is larger than ED, it means that this database sequence may be not similar to the query sequence. This database sequence can be omitted in the following SW computations. It is worth to note that a FD will be influenced by the sequence lengths between query and database sequences. When the length of a database sequence is longer than that of query sequence, a FD of these two sequences may be large due to their length difference. However, the possible subsequences in this database sequence may be similar to the query sequence. Hence, FDFS cannot be applied to the database sequence with a length longer than that of the query sequence in order to avoid the possible false negatives.

### 2.4. Related Works

Recently, many works have been proposed in the past to implement the SW algorithm on GPU. In the following, the brief descriptions of selected works have been made by considering the implementations and performance.

In 2006, Liu et al. [14] proposed the hardware implementation of the double affine Smith-Waterman (DASW) algorithm on a graphics card by using graphic API (OpenGL + GLSL). By only computing alignment scores, DASW achieved 24 M (millions or mega) DP cells per second on single NVIDIA GeForce 7800 GTX GPU card. Liu et al. [15] also presented an approach for the protein database scanning by using a graphics card (OpenGL + GLSL) to gain a high performance at the low cost. The proposed approach achieved more than 650 M cell updates per second (abbreviated to CUPS) on single NVIDIA GeForce 7800 GTX GPU card. Moreover, it ran 9 times and 15 times faster than SSEARCH [32] and OSEARCH [32], respectively. The above works are both proposed based on GPU by OpenGL (a GPGPU programming), not CUDA.

The SW-CUDA [16] precomputed a query profile stored in the TM to replace the query sequence and the substitution matrix. In SW-CUDA, each TD in a GD is used to do a SW computation by using the query profile and a database sequence. This process matched the definition of the ITE technique proposed by the literature [19]. Hence, SW-CUDA preordered the database sequences according to their lengths in order to balance the computing workload of each TD in a TB. The preordered database sequences are stored in the GM. For each SW computation, the alignment matrix is computed column by column and the similarity score is stored in the LM. The SW-CUDA only calculated the similarity score for a SW computation and achieved 1830 MCUPS (=1.8 giga-CUPS) and 3480 MCUPS (=3.48 GCUPS) on single and dual-NVIDIA GeForce 8800 GTX GPU cards, respectively, and it ran 2 to 30 times faster than any previous implementation of SW on GPU.

GSW algorithm [17] pointed out that the design of query profile is not suitable for GPU due to the limited size of TM on GPU. They still used the query sequence and the substitution matrix both stored in the CM to do the SW computations by using the ITE technique. They also proposed an efficient function with an ASCII code table to access the score in the substitution matrix. The database sequences are stored in the GM and the similarity scores are stored in the sM. The GSW algorithm ran 10 times faster than SSEARCH on single NVIDIA Tesla C870 GPU card; however, it is slower than Farrar's implementation [32]. Another SW algorithm [18] by using the ITE technique achieved 7.5 and 14.5 GCUPS on single and dual-NVIDIA GeForce 9800 GTX GPU cards, respectively.

In CUDASW++1.0 [19], the ITE technique means that each SW computation consisting of a pair of query and database sequences (denoted as a task) is assigned to one TD; the ITR technique means a task is assigned to one TB. Due to the limited memory on GPU, each TD can only process a pair of short sequences in a TB with many TDs by using the ITE technique. Conversely, a pair of long sequences can be processed by a TB with many TDs by using the ITR technique. However, the ITE technique can achieve better performance than the ITR technique due to more tasks concurrently executed on GPU. The alignment matrix is computed according to the diagonal direction in CUDASW++1.0. For the ITE technique, they also considered the effect of coalesced access in the GM. Therefore, a preordered database is also used in CUDASW++1.0 as the previous works above. However, these preordered database sequences should be rearranged and then stored in the GM. In CUDASW++1.0, a threshold is set to 3072 for the length of database sequence. If the length of database sequence is less than the threshold, the SW computation is done by the ITE technique, otherwise by the ITR technique. CUDASW++1.0 achieved an average performance of 9.5 GCUPS and 14.5 GCUPS on single NVIDIA GeForce GTX 280 GPU card and dual-NVIDIA GeForce GTX 295 GPU card, respectively. However, the lengths of few sequences in the test Swiss-Prot protein database (release 56.6) are larger than the threshold [23]. CUDASW++2.0 [20] further optimized the performance of CUDASW++1.0 based on the SIMT abstraction of CUDA-enabled GPUs and achieved 17 GCUPS and 30 GCUPS on single NVIDIA GeForce GTX 280 GPU card and dual-NVIDIA GeForce GTX 295 GPU card, respectively. CUDASW++3.0 [26] achieved the maximum performance of 119.0 and 185.6 GCUPS on single NVIDIA GeForce GTX 680 GPU card and dual-GPU GeForce GTX 690 GPU card, respectively.

The alignment matrix is computed according to the row direction in CUDA-SSCA#1 [21]. The SSCA#1 benchmark was used to evaluate 5 kernels that are various permutations of the SW algorithm, including saving the alignment results or not. In order to save the alignment result of a SW computation, CUDA-SSCA#1 needs to spend more memory space by using a traceback procedure. The HKA algorithm [22] processed the SW computations by using the ITE technique and achieved 1.13 times better than CUDASW++2.0 in terms of GCUPS. Hains et al. found that CUDASW++1.0 only achieved 1.5 GCUPS when comparing the same query and database sequences on single NVIDIA Tesla C1060 GPU card by using the ITR technique. Their ITR kernel [23] obtained better performance than that of CUDASW++1.0.

## 3. Methods

**Figure 2:**
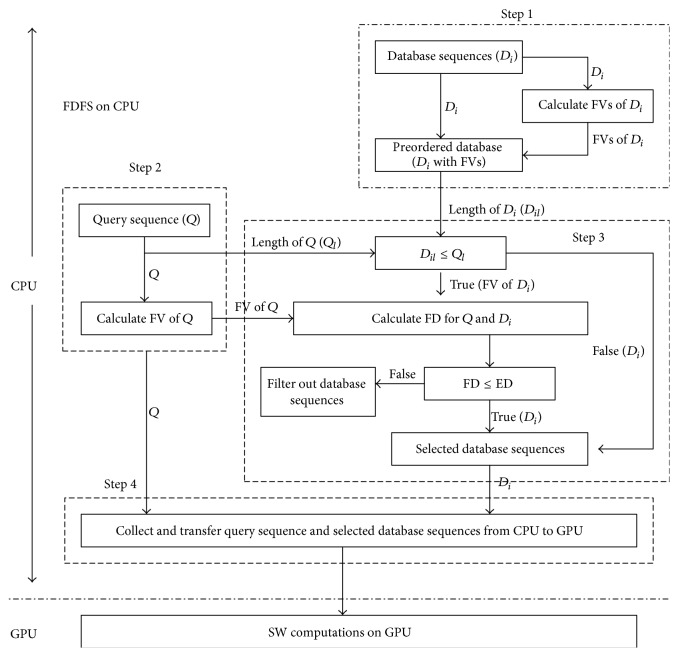
The flowchart of CUDA-SWfr.

The implementation of CUDA-SWfr can be divided into two parts: FDFS executed on CPU and the SW computations executed on GPU. The flowchart of CUDA-SWfr is shown in Figure 2. The details of these two parts are described in the following sections, respectively.

### 3.1. FDFS Executed on CPU

In CUDA-SWfr, there is a procedure to do FDFS on CPU. There are four steps in this procedure: (1) construct the preorder database sequences with FVs, (2) calculate the FV of query sequence, (3) calculate the FDs for each pair of query and database sequences, and (4) collect the query and selected database sequences and then they are transferred from CPU to GPU. In the following, these four steps are described in detail, respectively.

**Step 1 (construct the preorder database sequences with FVs).**

In the previous works in Section 2.4, the database sequences should be preordered according to their lengths due to the ITE technique. It is unnecessary for CUDA-SWfr by using the ITR technique. However, the following calculation of FD will be influenced by the sequence lengths between the query and database sequences as mentioned in Section 2.3. The database sequences are sorted and then stored in a database with their FVs in order to accelerate the computations of Step 3. The computation time of this step is omitted in the experimental test as the previous works in Section 2.4.

**Step 2 (calculate the FV of query sequence).**

When the biologists want to use CUDA-SWfr for a query sequence, the FV of query sequence is calculated in the run-time by using formula (3). Moreover, the length of query sequence is also recorded in a variable. The FV and length of query sequence will be used in Step 3.

**Step 3 (calculate the FDs for each pair of query and database sequences).**

As mentioned in Section 2.3, FDFS cannot be applied to the database sequence with a length longer than that of the query sequence in order to avoid the possible false negative. Hence, when a database sequence has the length longer than that of the query sequence, it needs to be compared in the second part of CUDA-SWfr and it is not necessary to calculate the FD with the query sequence. When the length of query sequence is short, this way will reduce a lot of time for Step 3. However, when a database sequence has the length shorter than or equal to the query sequences, the FD should be calculated by using formula (4) with the query sequence. When the calculated FD is larger than a threshold of ED, this database sequence is not selected according to formula (5). The threshold is set by the user. For the effect of FDFS, there are two factors. One is the length of query sequence and the other is the threshold of ED. When the length of query sequence is short, the lengths of most of database sequences may be longer than it, and the number of selected database sequences is large; in other words, the effect of FDFS is small. Similarly, when the threshold of ED is set to large, the FDs of most of database sequences may be smaller than this threshold, and then the effect of FDFS is small; see the experimental results in Section 4.

**Step 4 (collect the query and selected database sequences and then they are transferred from CPU to GPU).**

In this step, the FVs of query and selected database sequences and FDs of a pair of query and selected database sequences are not needed to be transferred from CPU to GPU. By FDFS, the transmission data (especially for the selected database sequences) can be reduced, and then the transmission time is decreased according to two factors mentioned above. Therefore, there are two advantages by using FDFS. One is to filter out the unnecessary alignments and the other is to reduce the transmission time. Moreover, the time by Steps 3 and 4 can be overlapped with the time of SW computations executed on GPU. After this step, the second part of CUDA-SWfr is used to do the SW computations on GPU.

### 3.2. SW Computations Executed on GPU

After the first part of CUDA-SWfr, the selected database sequences are stored in the GM and the query sequence is stored in the CM. When the length of query sequence is larger than the size of the CM, it is stored in the GM. In the Fermi architecture, it may be fast to access the query sequence in the GM due to the L2 caches. In CUDA-SWfr, the SW computations are made by using the ITR technique, and it means that the database sequences do not need to be rearranged before being stored in the GM. For the SW algorithm, a substitution matrix is needed. Hence, a substitution matrix is also stored in the CM. In order to access the score in a substitution matrix efficiently, the function with an ASCII code table presented by Striemer and Akoglu [17] is also used in CUDA-SWfr.

By the ITR technique, all TDs in a TB are used to do a SW computation; it is a dynamic programming algorithm. According to the previous works and the SIMT scheme on GPUs, there are eight implementation types of dynamic programming by using the ITR technique on GPU [33]. These eight implementation types are synchronous row single thread (SRST), synchronous row multiple threads (SRMT), asynchronous row single thread (ARST), asynchronous row multiple threads (ARMT), synchronous diagonal single thread (SDST), synchronous diagonal multiple threads (SDMT), asynchronous diagonal single thread (ADST), and asynchronous diagonal multiple threads (ADMT). CUDA-SWfr adopts the SRMT type of dynamic programming to implement a SW computation by an assignment method, in which one row is assigned to a TB until all rows are assigned.

For a SW computation in a TB, according to formulas (1) and (2) and the dependency shown in Figure 1, this process can be divided into two stages. In the first stage, the value of each cell (*H*(*i*, *j*)) in a row is calculated according to the upper cell, upper-left cell and 0. All cells in a row are calculated by all of TDs in a TB. Assume that the length of a row is *n* and the number of TDs in a TB is *t*; the values of (*n*/*t*) cells are calculated by a TD, where *n* is larger than *t* in general. However, these values are only intermediate values without considering the left cell according to formula (1) and (2). These values in a row are stored in the sM. In the second stage, only a TD in a TB is used to correct the value of each cell in a row by considering its left cell. These two stages will be done repeatedly until all rows are assigned to this TB. The highest value among all cells in all rows is the similarity score for a query and a selected database sequence. For a query sequence, all of similarity scores or the best score will be copied from the sM to the GM, and then they could be transferred from GPU to CPU according to the user's requirement. In CUDA-SWfr, the number of TDs in a TB is set to 256 and the number of TBs in a GD is equal to the number of selected database sequences (or partial of them in order to overlap the computation time between FDFS and SW computations).

The goal of this paper is to accelerate SW computations for the protein database search by using FDFS based on a CPU-GPU collaborative system. As the previous works [16–19] in the past, the alignment quality is not the consideration in this work. Only similarity scores are calculated for each pair of query and database sequences by using the SW algorithm. The query and selected database sequences can be aligned again by using other alignment algorithms or tools with specific substitution matrices in order to obtain the good alignment results or other simulation results, such as the homology modeling.

## 4. Experiment Results

In this work, CUDA-SWfr is implemented by C+CUDA on single NVIDIA Tesla C2050 GPU card, with 448 SPs cores and 3 GB GDDR3 RAM. The* Host* device is Intel Xeon E5506 CPU of 2.13 GHz with 12 GB RAM running Ubuntu 10.04 operation system.

In order to evaluate the effect of FDFS in CUDA-SWfr, the synthetic sequences are generated in this work. A test query sequence with a length of 1028, NP_001116291, is a human protein sequence downloaded from the NCBI website (http://www.ncbi.nlm.nih.gov/). Three subsequences are extracted randomly from it with the lengths of 256, 512, and 768, respectively. These three subsequences and the original query sequence are used as the query sequences in the following tests. Based on these query sequences, 12 synthetic databases are generated and used in the experimental tests. These 12 synthetic databases can be classified into 4 groups according to the lengths: 256, 512, 768, and 1028. For each group, there are three synthetic databases with 10000, 20000, and 40000 database sequences. For each database sequence in a group, it is generated according to the corresponding query sequence with the same length. For each synthetic database, it has database sequences with 0%~100% mutations of its corresponding query sequence. For example, assume that a synthetic database has 40000 database sequences and the length is 1028. There are 4000 database sequences with 0%~10% mutations of query sequence, 4000 database sequences with 10%~20% mutations of query sequence, and so on. When we want to generate one database sequence with 0%~10% mutations of query sequence, a number *t* is randomly selected between 0 (1028 × 0%) and 102 (1028 × 10%). If *t* is 57, it means that 57 positions in the query sequence are randomly selected at first, and then the residues in these 57 positions are changed to others by adding a constant (also randomly selected) with its original ASCII code. This work is done repeatedly until 40000 database sequences are generated for this synthetic database. In the following tests, the threshold of FDFS is the sequence identity. In other words, if a threshold is set to 90%, it means that the selected database sequence may be very similar to the query sequence.

**Figure 3:**
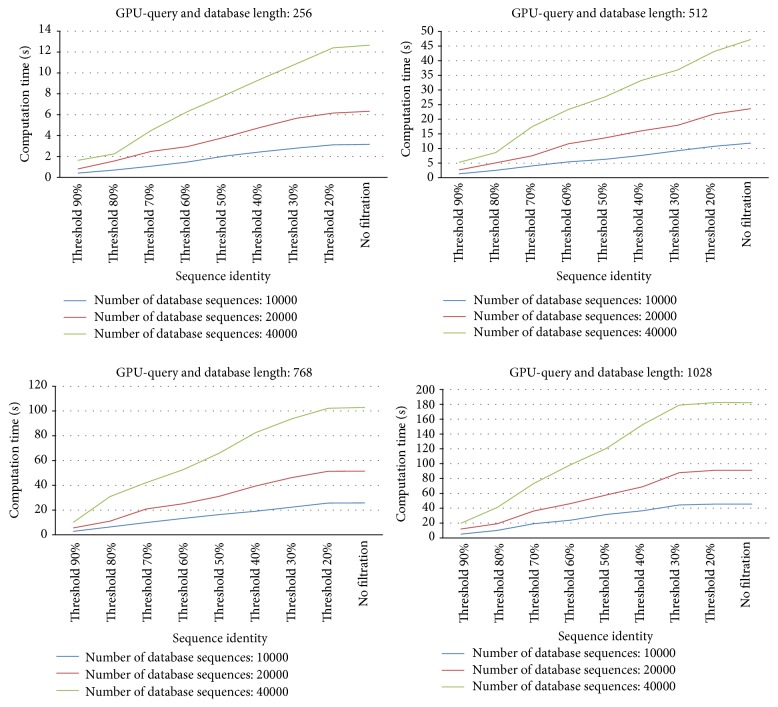
The overall computation time by CUDA-SWfr with various thresholds for 12 synthetic databases.

Figure 3 shows the overall computation time (unit: second) by CUDA-SWfr with various thresholds for 12 synthetic databases. The threshold “None” means the SW computations by CUDA-SWfr without FDFS. From Figure 3, the computation time decreases when the threshold increases for 12 synthetic databases. When the threshold is set to 90%, the computation time by CUDA-SWfr is fastest for all of 12 synthetic databases. From Figure 3, it also shows that the computation time increases when the sequence length or the number of database sequences increases.

**Figure 4:**
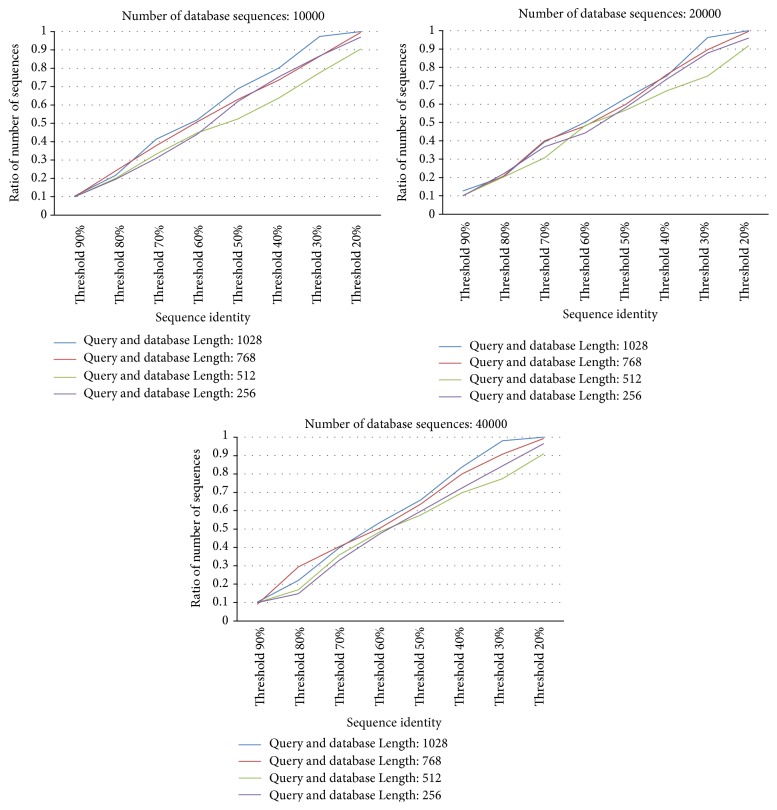
Ratio (%, number of selected database sequences/number of original database sequences) for 12 synthetic databases with various thresholds.

In order to observe the relationship between the threshold and the number of selected database sequences, Figure 4 shows the ratio (%) by dividing the number of selected database sequences by the number of original database sequences for 12 synthetic databases with various thresholds. From Figure 4, the ratio decreases when the threshold increases for 12 synthetic databases. When the threshold is 90%, in Figure 4, only 10% database sequences are selected for the SW computations on GPU for 12 synthetic databases. These results explain why the computation time by CUDA-SWfr is fastest when the threshold is set to 90%. From Figure 4, it also shows that the ratio is not influenced by the number of database sequences for 12 synthetic databases. Besides, when the length of query sequence is equal to that of database sequences, the ratio is also not influenced by the sequence length for 12 synthetic databases. However, the ratio will be influenced by the sequence length when the length of query sequence is not equal to that of database sequences. As mentioned in Section 2.3, when the length of database sequence is larger than that of query sequence, this database sequence should be compared with the query sequence. Hence, when database sequences have various lengths, for a query sequence, the ratio will increase when the number of database sequences, each having a length larger than that of query sequence, increases.

**Figure 5:**
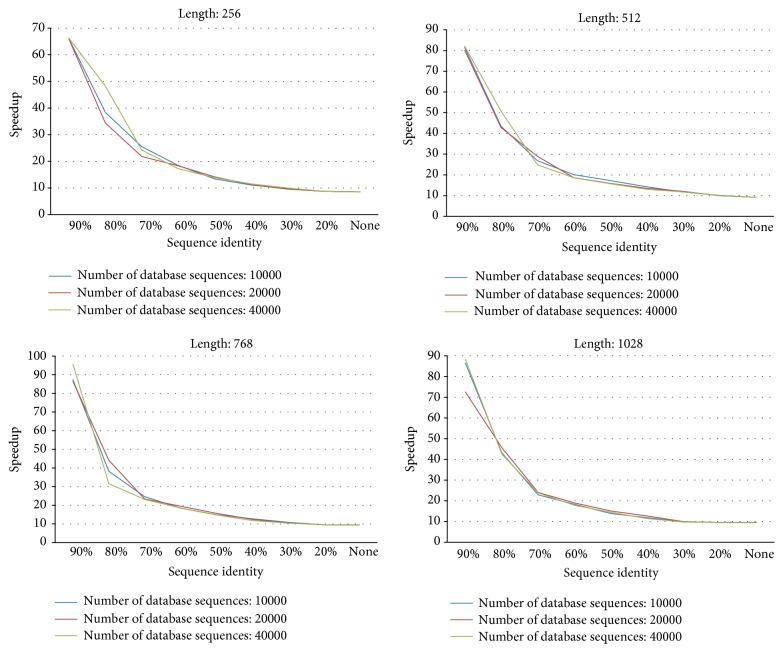
The overall speedup by comparing CUDA-SWfr with the CPU-based SW method under various thresholds for 12 synthetic databases.

Figure 5 shows the overall speedups by comparing CUDA-SWfr with the CPU-based SW method under various thresholds for 12 synthetic databases. From Figure 5, the overall speedups by CUDA-SWfr without FDFS are about 8.5~9.6 times. The best speedup by CUDA-SWfr with FDFS achieved 96 times. From Figure 5, the speedup increases when the threshold increases for 12 synthetic databases. From Figure 5, it also shows that the speedup is not influenced by the number of database sequences for 12 synthetic databases. Besides, when the length of query sequence is equal to that of database sequences, the speedup is also not influenced by the sequence length for 12 synthetic databases. The observations in Figure 5 match those of Figures 3 and 4. Unlike the previous works that use the GCUPS to evaluate the performance, CUDA-SWfr only considered the computation time and the speedups. There are two reasons. First, the overall computation workload (DP cells) by CUDA-SWfr is difficult to be estimated due to filtering out the unnecessary alignments by FDFS. Second, the overall computation time by CUDA-SWfr consisted of FV and FD calculations (Steps 2 and 3 in Section 3.1), SW computations (Section 3.2), and transmission time (Step 4 in Section 3.1 and Section 3.2). Hence, it is hard to estimate the correct value of GCUPS. Assume that the overall computation time is spent to calculate overall cells. CUDA-SWfr achieved the peak performance of 2.133 GCUPS by using the ITR technique.

**Figure 6:**
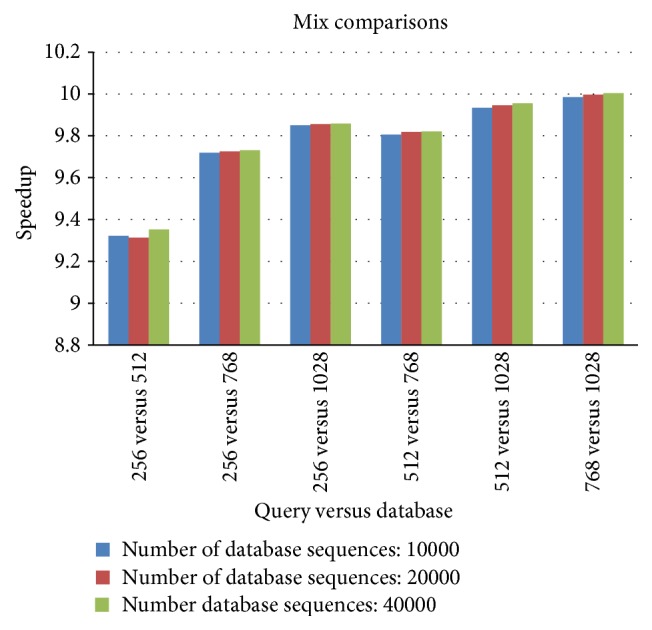
The overall speedup by comparing CUDA-SWfr with the CPU-based SW method for various combinations.

When the length of query sequence is less than the length of database sequences, Figure 6 shows the overall speedup by comparing CUDA-SWfr with the CPU-based SW method for various combinations. The query sequence of length 256 was used to compare with the synthetic database of lengths 512, 768, and 1028, respectively; the query sequence of length 512 was used to compare with the synthetic database of lengths 768 and 1028, respectively; the query sequence of length 768 was used to compare with the synthetic database of length 1028. The overall speedups by CUDA-SWfr in Figure 6 are about 9.3~10 times. Since the length of database sequence is larger than that of query sequence, all of database sequences should be compared with the query sequence. These results are similar to those (8.5~9.6 times) by CUDA-SWfr without FDFS as shown in Figure 3. From Figure 6, the speedup increases when the length of database sequences increases. The reason is that the overall computation workload increases when the length of database sequences increases.

In this work, we did not use the real biological sequences to evaluate the effect of FDFS. There are two reasons. The first one is the lengths of real biological sequences are various. In order to avoid the possible false negative, FDFS cannot be applied to the database sequence with a length longer than that of the query sequence. Hence, it is hard to choose the suitable set of real biological sequences and then to prove the relationships of ratio-threshold and ratio-speedup on GPU. The second one is the range of similarity scores among real biological sequences is large. In practice, it shows that FDFS scheme is necessary to filter out the unnecessary alignments. However, in the experimental tests, it is hard to choose the suitable set of real biological sequences to prove the relationships of threshold-speedup on GPU.

## 5. Conclusions

In this paper, we presented a GPU-based SW alignment method, CUDA-SWfr, with FDFS by using the ITR technique. The FDFS is executed by the CPU capability to filter out the unnecessary alignments and the SW computations are made by the GPU capability. From the experimental tests, CUDA-SWfr ran 9.6 times faster than the CPU-based SW method without FDFS; it ran 96 times faster than the CPU-based SW method with FDFS. The effect by FDFS is influenced greatly by the threshold. When the threshold as the sequence identity is large, the number of selected database sequences is small which means that the effect by FDFS is visible. In other words, the effect by FDFS can be omitted with a small threshold. Besides, the effect by FDFS also could be influenced by the number of database sequences; each has a length larger than that of query sequence. CUDA-SWfr is suitable for the protein database search with a lot of database sequences, and the proposed FDFS can be integrated into other research works, such as Huang et al. [34] and Feng et al. [35], in recent years.
